# Prevalence and Characterization of Gastroenteritis Viruses among Hospitalized Children during a Pilot Rotavirus Vaccine Introduction in Vietnam

**DOI:** 10.3390/v15112164

**Published:** 2023-10-27

**Authors:** Chu Thi Ngoc Mai, Le Thi Khanh Ly, Yen Hai Doan, Tomoichiro Oka, Le Thi Phuong Mai, Nguyen Tu Quyet, Tran Ngoc Phuong Mai, Vu Dinh Thiem, Lai Tuan Anh, Le Van Sanh, Nguyen Dang Hien, Dang Duc Anh, Umesh D. Parashar, Jacqueline E. Tate, Nguyen Van Trang

**Affiliations:** 1 National Institute of Hygiene and Epidemiology, Hanoi 100000, Vietnam; chumai93@gmail.com (C.T.N.M.); klylebio@gmail.com (L.T.K.L.); phuongmai.0626@gmail.com (T.N.P.M.); vdt@nihe.org.vn (V.D.T.);; 2 Center for Emergency Preparedness and Response, National Institute of Infectious Diseases, Tokyo 208-0011, Japan; 3 Department of Virology II, National Institute of Infectious Diseases, Tokyo 208-0011, Japan; 4 Nam Dinh Center for Disease Control, Nam Dinh 420000, Vietnam; 5 TT Hue Center for Disease Control, Hue, Thua Thien Hue 530000, Vietnam; 6 Center for Research and Production of Vaccines and Biologicals, Hanoi 100000, Vietnam; 7 Centers for Disease Control and Prevention, Atlanta, GA 30333, USA

**Keywords:** gastroenteritis viruses, rotavirus vaccine, Rotavin-M1, Vietnam, rotavirus, norovirus, sapovirus, astrovirus

## Abstract

Rotavirus (RV), norovirus (NoV), sapovirus (SaV), and human astrovirus (HAstV) are the most common viral causes of gastroenteritis in children worldwide. From 2016 to 2021, we conducted a cross-sectional descriptive study to determine the prevalence of these viruses in hospitalized children under five years old in Nam Dinh and Thua Thien Hue provinces in Vietnam during the pilot introduction of the RV vaccine, Rotavin-M1 (POLYVAC, Hanoi, Vietnam). We randomly selected 2317/6718 (34%) acute diarrheal samples from children <5 years of age enrolled at seven sentinel hospitals from December 2016 to May 2021; this period included one year surveillance pre-vaccination from December 2016 to November 2017. An ELISA kit (Premier Rotaclone^®^, Meridian Bioscience, Inc., Cincinnati, OH, USA) was used to detect RV, and two multiplex real-time RT-PCR assays were used for the detection of NoV, SaV and HAstV. The prevalence of RV (single infection) was reduced from 41.6% to 22.7% (*p* < 0.0001) between pre- and post-vaccination periods, while the single NoV infection prevalence more than doubled from 8.8% to 21.8% (*p* < 0.0001). The SaV and HAstV prevalences slightly increased from 1.9% to 3.4% (*p* = 0.03) and 2.1% to 3.3% (*p* = 0.09), respectively, during the same period. Viral co-infections decreased from 7.2% to 6.0% (*p* = 0.24), mainly due to a reduction in RV infection. Among the genotypeable samples, NoV GII.4, SaV GI.1, and HAstV-1 were the dominant types, representing 57.3%, 32.1%, and 55.0% among the individual viral groups, respectively. As the prevalence of RV decreases following the national RV vaccine introduction in Vietnam, other viral pathogens account for a larger proportion of the remaining diarrhea burden and require continuing close monitoring.

## 1. Introduction

Diarrhea in children may be caused by as many as 30 different agents consisting of viruses, bacteria, and parasites. Among these pathogens, rotavirus (RV), norovirus (NoV), sapovirus (SaV), human astrovirus (HAstV), and adenovirus (AdV) are the most common viral agents, which account for 70% of acute gastroenteritis (AGE) cases worldwide [1]. In particular, RV is the leading cause of AGE among hospitalized children. In countries that have introduced a rotavirus vaccine, AGE hospitalizations declined globally by 36% in the period from 2006 to 2019 [2]. In Vietnam, multiple RV vaccines have been available in the private market, including Rotarix^®^ (GlaxoSmithKline, London, UK) and RotaTeq™ (Merck Sharp and Dohme, West Point, PA, USA) since 2008), and Rotavin-M1 (POLYVAC, Hanoi, Vietnam) since 2012. However, RV vaccines have not yet been included in the National Immunization Programme of Vietnam.

NoV also accounts for a high frequency of infection among children with AGE, causing 677 million cases and 213,515 deaths each year, according to a 2015 study [3]. A recent meta-analysis revealed that the global prevalence of NoV among AGE cases from 1997 to 2021 ranged from 17% to 20%, highlighting the importance of this virus in human morbidity [4]. The NoV prevalence was 28% in Vietnam from 2012 to 2015, higher than in Indonesia (12.3% in 2015–2019) and China (15.4% in 2012–2017) [5,6,7]. Similarly, HAstV and SaV infections also contribute to diarrheal hospitalizations and outbreaks. Globally, HAstV prevalence among children with AGE was 4.2% (95% CI: 3.8–4.8%), with the most common genotype being HAstV-1 (59%) [8]. The prevalence of SaV globally was 3.4% among children with AGE, and genogroup I was the dominant type [9]. Several studies have suggested that the prevalences of NoV, SaV and HAstV have been significantly altered after RV vaccine introduction among children less than 5 years of age [10,11,12].

In this study, we determined the prevalences and characteristics of RV, NoV, SaV, and HAstV in children hospitalized with AGE in seven Vietnamese sentinel hospitals in Nam Dinh and TT Hue provinces to investigate if there was a shift in viral pathogen burden after a Rotavin-M1 vaccine pilot introduction in these regions from 2017 to 2020.

## 2. Materials and Methods

### 2.1. Study Design

A live-attenuated G1P [8] strain RV vaccine (Rotavin-M1), which was licensed for local use in 2012, was provided free of charge to all children starting from 2 months of age between December 2017 and November 2020 as part of a pilot project in four districts in Nam Dinh province, North Vietnam (Giao Thuy, Hai Hau, Xuan Truong, and Truc Ninh), and two districts in TT Hue province, Central Vietnam (Huong Tra, Phu Vang) [13]. This vaccine schedule consists of 2 doses for children from 2 months old, with an interval of at least 1 month between doses, and the last dose should be completed before 6 months of age. During our surveillance period, not all children were age-eligible to receive the rotavirus vaccine. Vaccine coverage for age-eligible diarrheal cases was 77% in Nam Dinh and 43% in Hue [13]. This study analyzed data from all cases, regardless of their vaccination status.

**Figure 1:**
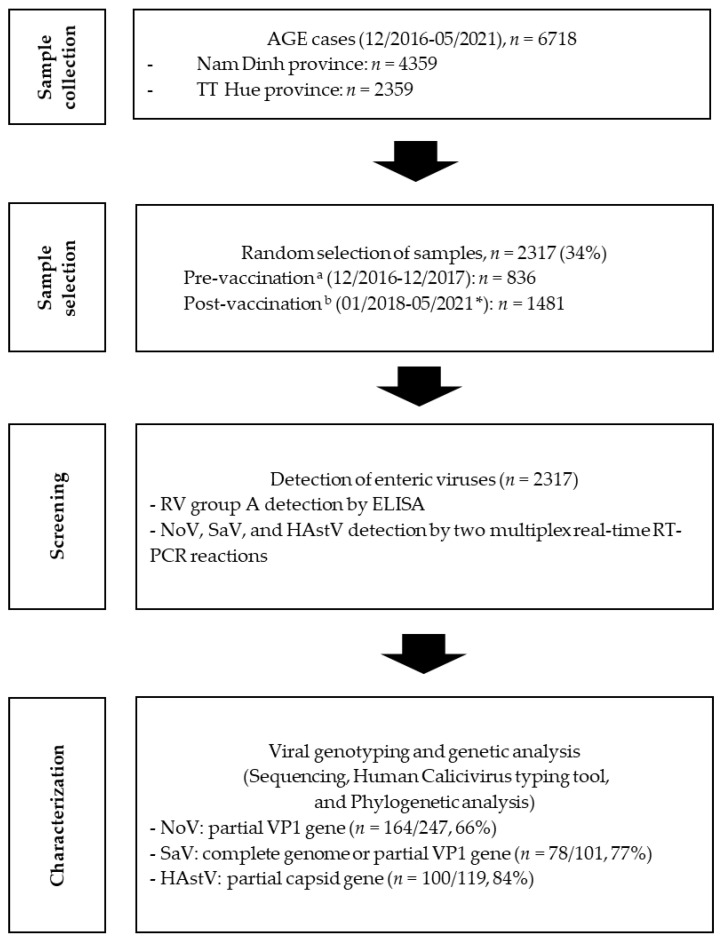
A flowchart of sample collection and testing (pre-vaccination period ^a^: from December 2016 to March 2018 for Nam Dinh province and from December 2016 to December 2017 for TT Hue province; post-vaccination period ^b^: from April 2018 to May 2021 for Nam Dinh province and from January 2018 to May 2021 for TT Hue province; * all samples collected in 2021 were included in the analyses).

Sentinel sites were set up in 6 district hospitals in the regions and in a central hospital in Hue to capture diarrheal cases. Fecal samples were collected from children under 5 years of age, who were admitted to the district hospitals in the aforementioned districts and in the Hue central hospital with a clinical diagnosis of AGE from December 2016 to May 2021. All stool samples were stored at −20 °C at these study sites until being shipped to the National Institute of Hygiene and Epidemiology (NIHE), where they were stored at −70 °C until the analyses. Between 30 and 50% of the samples were selected each year from 2016 to 2020 to test for the four viruses, RV, NoV, SaV, and HAstV. In 2021, all samples were selected for the analyses of these viruses, due to the small number of enrolled cases (Figure 1). Thus, in total, 2317 samples were analyzed for the 4 viruses.

### 2.2. Human Group A RV Detection via ELISA

Stool samples were tested for the presence of the human group A RV antigen using a commercial Premier Rotaclone^®^ kit (Meridian Bioscience, Inc., Cincinnati, OH, USA), according to the manufacturer’s instructions.

### 2.3. Nucleic Acid Extraction

Viral nucleic acids were extracted from 200 μL of 25% fecal suspension, using a QIAcube^®^ HT automatic extraction system and a *Cador*^®^ Pathogen 96 Qiacube^®^ HT kit (Qiagen, Hilden, Germany), according to the manufacturer’s instructions. Viral nucleic acids were eluted in 100 μL of the elution buffer and then stored at −70 °C.

### 2.4. Detection of NoV, SaV, and HAstV via Multiplex Real-Time RT-PCR

In the first multiplex real-time RT-PCR reaction for detecting NoV GI, GII, and GIV simultaneously, primers and probes were adopted from Farkas’s study [14]. In the second reaction for SaV and HAstV detection, the primers and probe described in Oka et al. and Logan et al. were utilized [15,16]. Notably, different fluorescence dyes were used. For all multiplex real-time RT-PCR reactions, we used a SuperScript™ III Platinum™ One-step qRT-PCR system (Thermofisher, Waltham, MA, USA). The real-time RT-PCR conditions were 50 °C for 30 min, followed by 95 °C for 15 min, then 45 cycles of 95 °C for 20 s, and 57 °C for NoV detection or 60 °C for SaV and HAstV detection for 60 s. Thermal cycling was performed on a Rotor-Gene Q (Qiagen^®^, Hilden, Germany), and raw data were analyzed using Rotor-Gene Q software version 2.1.0.

### 2.5. Sequence and Phylogenetic Analyses

All SaV- and HAstV-positive specimens (101 and 119 specimens, respectively) and 50% of NoV-positive specimens (247 specimens) were randomly selected for genotyping. Regarding NoV, G1SKF/G1SKR and G2SKF/G2SKR primers [17] were used to amplify the partial VP1 gene regions of the GI and GII genotypes, 330 bp and 343 bp, respectively. Specimens positive for SaV were full-genome-sequenced using next-generation sequencing (NGS) at the National Institute of Infectious Diseases (NIID), Japan. Alternatively, partial VP1 gene regions were sequenced using SLV5317/SLV5749 primers (434 bp) [18] or M13F-SaV 1245Rfwd/M13R-SV-G1-R, M13R-SV-G2-R, M13R-SV-G4-R, and M13R-SV-G5-R [19] if NGS failed. Both NoV and SaV forward primers (G1SKF, G2SKF, SLV5317, and M13F-SaV 1245Rfwd) were designed for the RdRp region. For HAstV, a partial capsid gene (719 bp) was amplified using preCap1/82b primers [18]. The sequences were analyzed using Geneious software version 11.1.5. The genogroups, genotypes, and variants of NoV and SaV were established based on the Human calicivirus typing tool website of the Norovirus Laboratory Surveillance Network (https://calicivirustypingtool.cdc.gov/, accessed on 25 October 2023). The classification of HAstV was based on phylogenetic analysis, including the reference strains from GenBank representing different HAstV genotypes [20]. All the selected sequences were aligned using the MAFFT multiple sequence alignment program version 7 [21]. A maximum likelihood tree was constructed using the Kimura two-parameter method, with bootstrapping of 1000, using MEGA X software, version 10.2.6 [22].

### 2.6. Statistical Analyses

We analyzed and compared the prevalences of the viruses before and after vaccination using the chi-square test. Variables with a *p* value ≤ 0.05 were considered statistically significant. Data were analyzed using SPSS V22.0 (IBM, New York, NY, USA).

### 2.7. Ethical Issues

This research was approved by the Ethics Committee of the NIHE (number: IRB-VN01057-19/2016). Parents/legal guardians of all children in the study approved and signed the consent forms for their children to participate. All information on these participants, as well as samples, were kept confidential. The SaV genome sequence analysis was independently approved by the Ethics Committee of the NIID.

### 2.8. Accession Numbers

These Vietnam HAstV sequences were deposited in GenBank, with accession numbers from OR670604 to OR670615 and from OR690931 to OR690946.

## 3. Results

### 3.1. Distribution of Enteric Viral Infections

**Figure 2:**
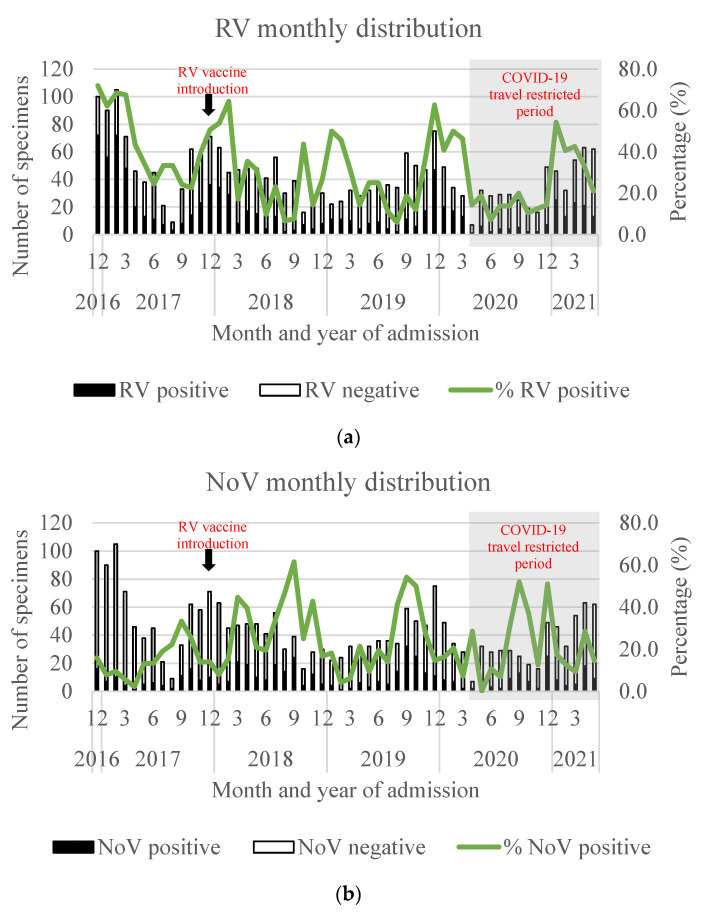

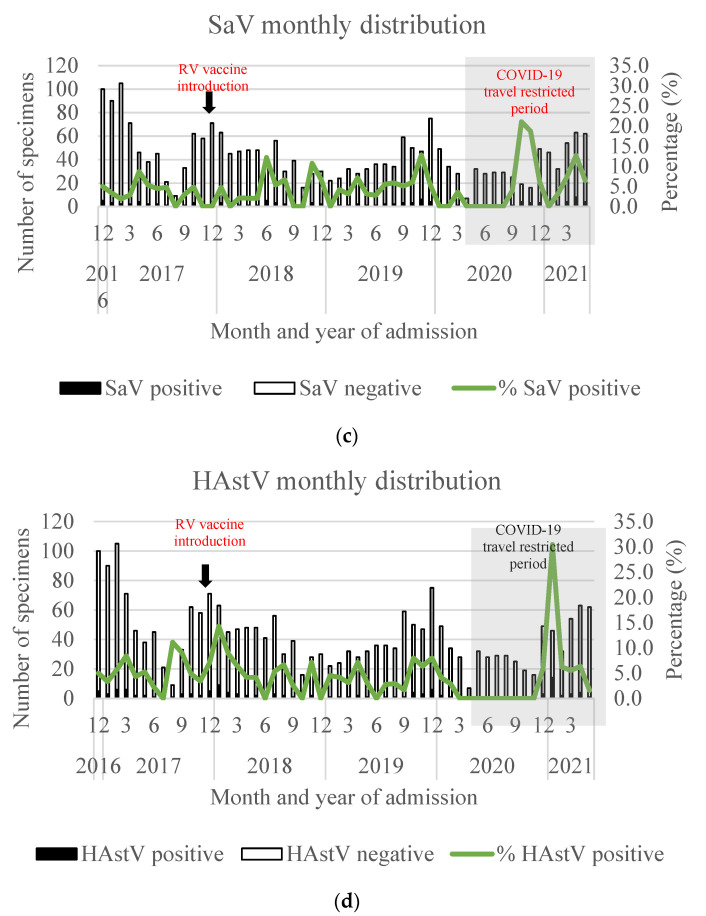
Monthly distribution of RV- (**a**), NoV- (**b**), SaV- (**c**), and HAstV- (**d**) positive specimens in Nam Dinh and TT Hue provinces. Black arrows show the time point of RV vaccine introduction (December 2017), and gray boxes show the COVID-19-restricted period (April 2020 to May 2021).

Among the 6718 samples collected during the period from 2016 to 2021, 1613 (37%) and 704 (30%) samples were randomly selected from 4359 and 2359 samples in Nam Dinh and TT Hue provinces, respectively, for detection of these four enteric viruses. RV positivity peaked from October to March. The peak of NoV-positive cases (between August and October) sharpened in the years following RV vaccine introduction. The numbers of SaV- and HAstV-positive cases were low, and the seasonal distribution of these viruses could not be clearly observed (Figure 2).

Comparing the pre- and post-vaccination periods, the detection rate of single RV infection dropped from 41.6% to 22.7% (range 21.4–24.9%) (*p* < 0.0001). In contrast, the NoV positivity more than doubled from 8.8% to 21.8% (range 17.0–25.4%) (*p* < 0.0001). Of note, the NoV-positive rate increased among the RV-negative group (9.4% vs. 22.6%, *p* < 0.0001), while it remained unchanged in the RV-positive group (3.4% vs. 3.0%, *p* = 0.5876). SaV and HAstV positivity increased from 1.9% to 3.4% (*p* = 0.03) and from 2.1% to 3.3% (*p* = 0.09), respectively (Table 1).

**Figure 3:**
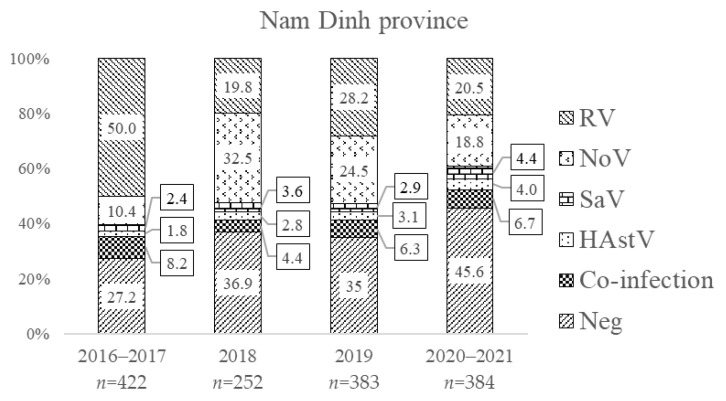

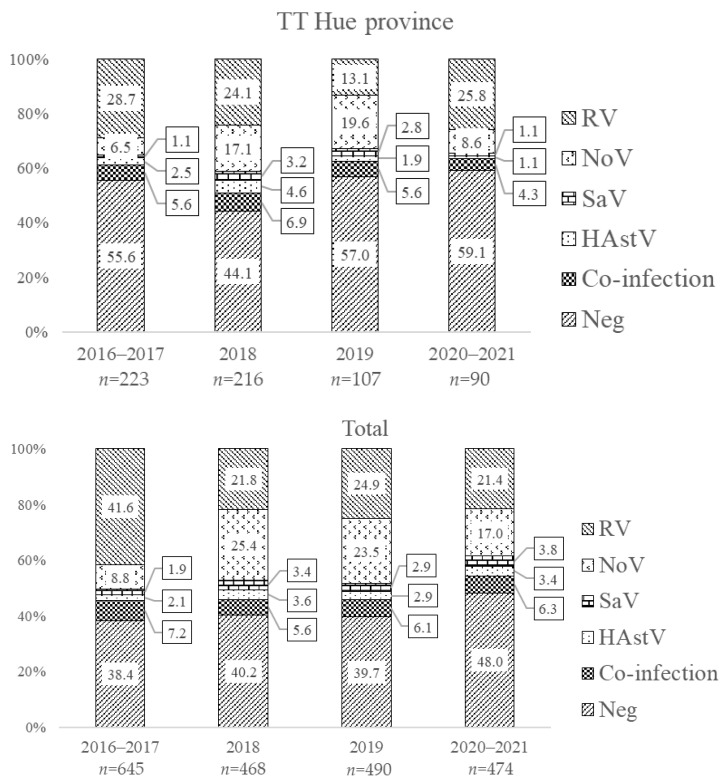
Frequency of RV, NoV, SaV, and HAstV single infections and co-infections in Nam Dinh and TT Hue provinces by year of admission.

The distributions of the four enteric viruses (including single and multiple infections) by year of admission are illustrated in Figure 3. Prior to vaccine introduction, single infection with RV was 50.0% and 28.7% in Nam Dinh and TT Hue, respectively. The prevalence of NoV was 15.1% and 11.5% in these two regions, of which single NoV infection only appeared in 10.4% and 6.5% of samples, respectively; the remaining (4.7–5.0%) NoV infections were found mixed with other viruses. In the first year after vaccine introduction (2018), single-infection RV positivity fell to 19.8% and 24.1%, respectively, in Nam Dinh and TT Hue. In the following years, RV prevalence went up to 28.2% in 2019 and 20.5% in 2020–2021 in Nam Dinh, while decreasing to 13.1% in 2019 and then increasing to 25.8% in 2020–2021 in TT Hue. Meanwhile, the single-infection NoV detection rate increased from 10.4% to 32.5% in Nam Dinh and from 6.5% to 17.1% in TT Hue one year after vaccine introduction. During this time, NoV became as dominant as RV and increased in detection as a co-infection, unlike prior to vaccine introduction. Similar patterns of infection for RV and NoV continued through the second and third years after vaccine introduction.

In Nam Dinh province, the prevalence of SaV and HAstV increased slightly from 2018 to 2021. This pattern was not as clear in TT Hue and was disrupted during the COVID-19 pandemic (Figure 3). Interestingly, in the years following vaccine introduction, the proportion of samples in which none of these four viruses could be found increased.

### 3.2. Co-Infections with Enteric Viruses

During the study period, 154/2317 (6.6%) cases were co-infected with any combination of the four viruses. Co-infection prevalence ranged between 7.8% before RV vaccine introduction and 6.0% after RV vaccine introduction. Co-infection with up to three enteric viruses was found. Co-infection of RV/NoV was the most common (47.7% and 51.7% of multiple-infection cases in the pre- and post-vaccination periods), followed by the co-infection patterns of RV/HAstV and RV/SaV. Furthermore, the prevalence of NoV co-infections increased after vaccine introduction (Table 2).

### 3.3. Genetic Characteristics of Enteric Viral Infections

In this study, 164/247 (66%), 78/101 (77%), and 100/119 (84%) of NoV, SaV, and HAstV samples were successfully sequenced and available for genotyping. Regarding the NoV genotype, one genotype of GI (GI.3) and seven genotypes of GII were detected, while no GIV was detected in this study. Genotype GII.4 was the most common (57.3%), of which variant GII.4 Sydney accounted for 91/94; the remaining variants were GII.4 Hong Kong (2/94) and GII.4 Yerseke (1/94). Other genotypes, including GII.3 (42/164, 25.6%), GII.2 (15/164, 9.1%), GII.6 (3/164, 1.8%), GII.7 (3/164, 1.8%), GII.10 (1/164, 0.6%), and GII.17 (2/164, 1.2%), were observed.

The genetic characteristics of the capsid gene from SaV-positive samples showed a higher level of genotype diversity. A total of ten different SaV genotypes were identified, including four GI genotypes (GI.1, GI.2, GI.5, and GI.6), four GII genotypes (GII.1, GII.2, GII.3, and GII.5), the GIV.1 genotype, and the GV.1 genotype. Among these, GI.1 and GII.1 were the most frequently detected in 25/78 (32.1%) and 20/78 (25.6%), respectively. Two samples contained two different SaV genotypes (GI.5 + GII.3 and GII.2 + GV.1).

**Figure 4:**
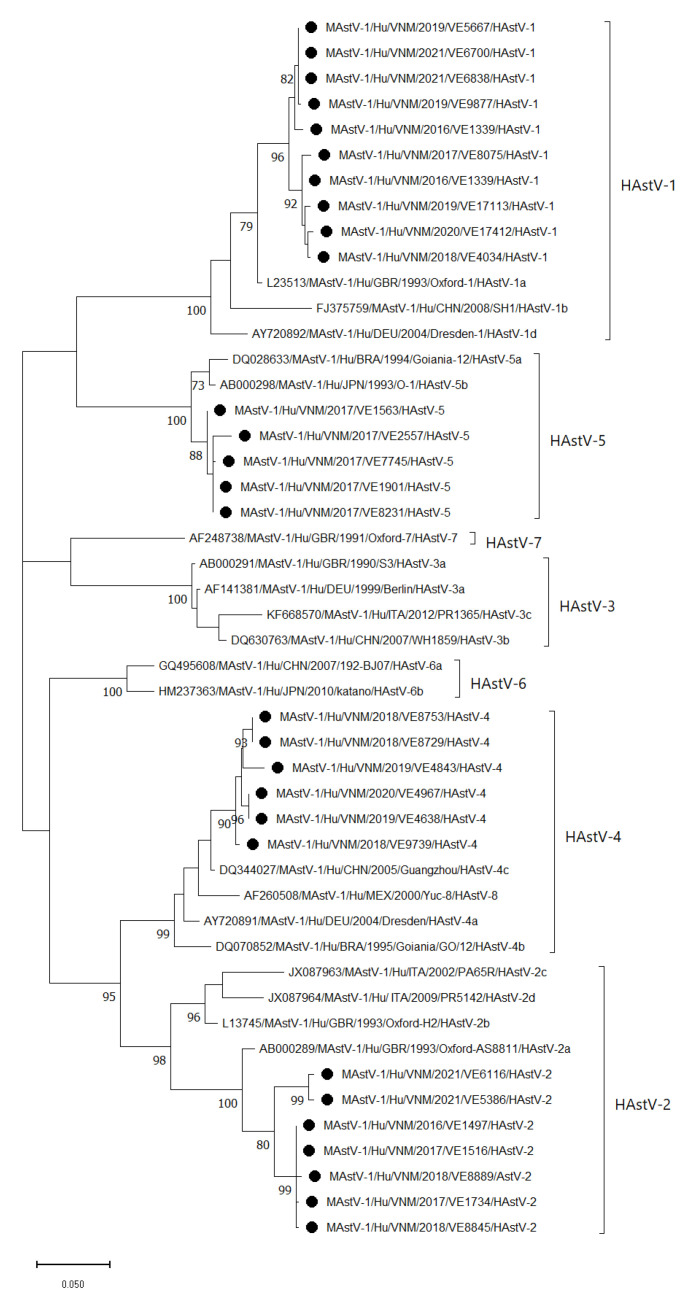
Maximum likelihood phylogenetic tree for partial capsid genes of HAstV strains detected in Vietnam (●) from 2016 to 2021. The scale bar and branch lengths represent the nucleotide substitutions per site. Bootstrap values of over 70 are presented. The following strains were used as reference strains: HAstV-1 (accession numbers: L23513, FJ375759, AY720892), HAstV-2 (accession number: AB000289, L13745, JX087963, JX087964), HAstV-3 (accession number: AB000291, AF141381, DQ630763, KF668570), HAstV-4 (accession number: AY720891, DQ070852,DQ344027), HAstV-5 (accession number: DQ028633, AB000298), HAstV-6 (accession number: GQ495608, HM237363), HAstV-7 (accession number: AF248738), and HAstV-8 (accession number: AF260508). Only the selected 28 strains out of 100 HAstV sequences from Vietnam were included in the phylogenetic tree (accession numbers from OR670604 to OR670615 and from OR690931 to OR690946).

A total of 100 capsid gene fragments of HAstV-positive samples were successfully amplified and sequenced (the 481-nucleotide region in the capsid). Genetic analysis revealed four circulating genotypes: HAstV-1, 2, 4, and 5. Further subtyping based on phylogenetic analysis showed that there were 55/100 HAstV-1, lineage a (55.0%); 19/100 HAstV-2, lineage a (19.0%); 19/100 HAstV-4, lineage c (19.0%); and 7/100 HAstV-5, lineage c (7.0%). The Vietnamese HAstV-1 sequences shared 95.5–100% nucleotide identity among themselves and up to 99.6% identity with other G1 sequences from GenBank. A total of 28 sequences, including 10 HAstV-1, 7 HAstV-2, 6 HAstV-4, and 5 HAstV-5 sequences, were included in the phylogenetic tree (Figure 4).

## 4. Discussion

In this study, the single RV infection rate in two provinces before vaccine introduction was 48.2%, which is comparable to the 46.7% reported in another study from 2012 to 2015 [23]. After rotavirus vaccine introduction, the RV detection rate among hospitalized diarrheal cases dropped ~50%, consistent with the 57% vaccine effectiveness of Rotavin-M1 in Vietnam [13]. The decline in RV prevalence seen in our study follows the global trend of a reduction in RV prevalence among hospitalized children <5 years old, from 40% during the pre-vaccination period to 20% during the 4 years after vaccination [2]. The genotype description for RV was previously published [13]. In brief, this vaccine strain is the G1P [8] genotype, but none of the G1 strains found in diarrheal children matched the vaccine strain. This determination was made by comparing the VP7 and VP4 gene sequences of the strains in the samples to the vaccine strain, revealing differences of 8–9.3% in the VP7 gene sequence and 2.5% in the VP4 sequence.

Following RV vaccine introduction, there have been several reports about the increasing prevalence of NoV causing AGE hospitalizations in the USA, Nicaragua, and Peru [24,25,26]. In our study, the prevalence of NoV more than doubled during the post-vaccine introduction period, and NoV became as predominant a viral pathogen as RV among AGE cases. The proportion of AGE hospitalizations due to NoV in Nam Dinh province was similar to that in another study in Vietnam from 2012 to 2015 (yearly range from 23.4 to 33.0%) [5]. The prevalence in TT Hue province showed similarities, with an estimated 17% NoV prevalence among acute gastroenteritis cases in developing countries, based on a meta-analysis of 178 articles from 1990 to 2016 [27]. This prevalence is lower than that found in previous studies (20.6–36%), which may be due to the difference in case admissions between district hospitals (this study) and major hospitals (previous studies) [5,28,29]. Genogroup GII is the predominant NoV group circulating in Vietnam, China, and other Asian countries. A study in Hanoi and Khanh Hoa province from 2012 to 2015 indicated that 86% of the NoV cases belonged to the GII genogroup, in which GII.4 and GII.3 accounted for 55% and 22%, respectively [5]. In Vietnam, in addition to the GII.4 Sydney predominance in 2013, other GII.4 variants were also observed. Variant GII.17 was detected at low frequency, though this variant emerged in China and Japan in 2014–2015 [5,30,31].

The SaV prevalence in this study was 4.6%, which is higher than that found in a previous study (1.4% in a single hospital in Hanoi) in the period 2007–2008 [28]. A greater diversity of genotypes of SaV was also recorded. This study observed genogroups GIV and GV of SaV, though the proportions were low. There were 11 genotypes of SaV belonging to four genogroups detected in Nam Dinh and TT Hue provinces in this study. The genotype diversity did not seem to be significantly different between the pre- and post-vaccination periods. SaV GI.1 and GII.1 accounted for 34.8% and 23.2% of cases, respectively. The higher diversity observed in our analysis may be due to the larger number of hospital sites involved in the surveillance and the inclusion of sites from two distinct provinces. A study in Vietnam (2007–2008) that included a single hospital detected three genotypes: GI.1, GI.2, and GII.1, while another study in Japan from 2014 to 2017 reported six SaV circulating genotypes: GI.1, GII.1, GIV.1, GI.2, GI.3, and GII.3, of which the proportion of GI.1 (83.3%) was higher than that in most other studies [28,32]. Genotypes GI.1 and GI.2 were the predominant circulating strains in other Asian countries and globally [33,34].

In Vietnam, the prevalence of HAstV in Nam Dinh province was higher than in TT Hue province in this study and in Thai Binh province in a study from 2011 to 2012 (2.4%), but lower than in Ho Chi Minh City during 2005–2006 (13.9%) [35,36]. HAstV-1 and 2 were circulating during the study period from 2016 to 2021 and HAstV-4 was detected in 2018–2020, whereas HAstV-5 was observed in 2017 only, at all study sites. HAstV-5 was also detected in Thailand during 2000–2011 and 2017–2020, and in China in 2017–2018, with a low proportion [33,37,38,39]. However, this HAstV-5 genotype was also found in Nigeria from 2015 to 2017 [40]. Studies in the periods 2002–2003 and 2005–2006 in the south of Vietnam found that all HAstV samples belonged to HAstV-1 [35,41]. In Vietnam, there was a difference between the HAstV-1 lineage detected in this study (HAstV-1a) and that in the aforementioned studies (HAstV-1d), suggesting that the most common lineage has been replaced. It is intriguing to note that all the detected HAstV strains in this study exhibited distinct branches when compared to the reference strains. This finding highlights the potential genetic diversity of Vietnamese HAstV. In light of these results, we intend to conduct further studies to explore the genetic diversity and evolution of Vietnamese HAstV at the whole-genome level.

Our study has limitations. Firstly, during the third study period following the introduction of the vaccine, we observed the impact of COVID-19 travel restrictions (April 2020 through May 2021) on the number of collected diarrhea cases. This impact was primarily due to a significant reduction in total hospital admissions and, to some extent, a shortage of medical staff who were engaged in SARS-CoV-2-related work. With children staying at home during this period, the frequency of childhood diseases could have declined. Moreover, changes in healthcare-seeking habits resulting from these restrictions posed a challenge in gathering a sufficient number of cases for analysis. Additionally, the high personal hygiene standards recommended by authorities, such as hand washing, effectively prevents the spread of enteric viruses; therefore, the estimated frequencies of these viruses would be biased. In our study, the numbers of untypable samples were still high (16–34%) for all three viruses due to the low copy numbers of the pathogens, reducing the amplification efficiency of the VP1 region used for sequencing and genotyping. Lastly, we also did not perform RdRp P-typing for NoV; therefore, we could not identify recombinant NoV strains, even though these are occurring at increasing frequencies in Vietnam.

## 5. Conclusions

In conclusion, this is the first study on the prevalences of NoV, SaV, and HAstV since the RV vaccine was introduced into routine use on a pilot scale in Vietnam. We revealed that the prevalences of other viral pathogens, especially NoV, increased post-vaccine introduction. These findings highlight the necessity to continue epidemiological surveillance of these pathogens after nationwide RV vaccine use in Vietnam.

## Figures and Tables

**Table 1:** The prevalence of single RV, NoV, SaV, and HAstV infection and co-infection in Nam Dinh and TT Hue provinces from 2016 to 2021.

Province		Nam Dinh			TT Hue			Total		
		Pre-Vaccination	Post-Vaccination	*p*-Value	Pre-Vaccination	Post-Vaccination	*p*-Value	Pre-Vaccination	Post-Vaccination	*p*-Value
AGE * cases	n	548	1065		356	416		904	1481	
RV single	n	274	246	*p* < 0.0001	102	90	*p* = 0.02	376	336	*p* < 0.0001
	%	50.0%	23.1%		28.7%	21.6%		41.6%	22.7%	
NoV single	n	57	257	*p* < 0.0001	23	66	*p* < 0.0001	80	323	*p* < 0.0001
	%	10.4%	24.1%		6.5%	15.9%		8.9%	21.8%	
SaV single	n	13	39	*p* = 0.16	4	11	*p* = 0.13	17	50	*p* = 0.03
	%	2.4%	3.7%		1.1%	2.6%		1.9%	3.4%	
HAstV single	n	10	36	*p* = 0.07	9	13	*p* = 0.62	19	49	*p* = 0.09
	%	1.8%	3.4%		2.5%	3.1%		2.1%	3.3%	
Coinfection	n	45	64	*p* = 0.10	20	25	*p* = 0.81	65	89	*p* = 0.25
	%	8.2%	6.0%		5.6%	6.0%		7.2%	6.0%	

* AGE: acute gastroenteritis.

**Table 2:** Patterns of co-infection among viral gastroenteritis pathogens in Nam Dinh and TT Hue provinces from December 2016 to May 2021 during a pilot RV vaccine introduction.

Co-Infection Pattern ^a^	Pre-Vaccination 2016–2017 ^b^		Post-Vaccination 2018–2021 ^c^	
	*n*	%	*n*	%
RV + NoV + (SaV/HAstV)	31	47.7	46	51.7
RV + SaV	9	13.8	15	16.9
RV + HAstV	19	29.2	14	15.7
NoV + HAstV/SaV	5	7.7	13	14.6
RV + SaV/HAstV	1	1.5	1	1.1
Total	65	100	89	100

^a^: The “+” sign indicates co-detection, the “/” sign indicates co-infection with either of the pathogens in the brackets. ^b^: From December 2016 to March 2018 in Nam Dinh province and from December 2016 to December 2017 in TT Hue province. ^c^: From April 2018 to May 2021 in Nam Dinh province and from January 2018 to May 2021 in TT Hue province.

## Data Availability

Data sharing inquiries should be directed to the authors.
